# Latin American cities with higher socioeconomic status are greening from a lower baseline: evidence from the SALURBAL project

**DOI:** 10.1088/1748-9326/ac2a63

**Published:** 2021-10-19

**Authors:** Yang Ju, Mika Moran, Xize Wang, Ione Avila-Palencia, Andrea Cortinez-O’Ryan, Kari Moore, Anne Dorothée Slovic, Olga L Sarmiento, Nelson Gouveia, Waleska Teixeira Caiaffa, Guilherme Aparecido Santos Aguilar, Denise Marques Sales, Maria De Fatima Rodrigues Pereira De Pina, Débora Moraes Coelho, Iryna Dronova

**Affiliations:** 1 Institute of Urban and Regional Development, University of California, Berkeley, CA, United States of America; 2 Department of Real Estate, National University of Singapore, Singapore, Singapore; 3 Urban Health Collaborative, Dornsife School of Public Health, Drexel University, Philadelphia, PA, United States of America; 4 Department of Physical Education, Sports and Recreation, Universidad de La Frontera, Temuco, Chile; 5 Environmental Health Department, School of Public Health, University of São Paulo, São Paulo, Brazil; 6 School of Medicine, Universidad de los Andes, Bogota, Colombia; 7 Departamento de Medicina Preventiva, Faculdade de Medicina da Universidade de São Paulo, São Paulo, Brazil; 8 Observatory for Urban Health in Belo Horizonte, School of Medicine, Federal University of Minas Gerais, Belo Horizonte, Brazil; 9 Instituto de Comunicação e Informação Científica e Tecnológica em Saúde, Fundação Oswaldo Cruz—ICICT/FIOCRUZ, Rio de Janeiro, Brazil; 10 Department of Environmental Science, Policy, and Management, Department of Landscape Architecture and Environmental Planning, University of California, Berkeley, CA, United States of America; 11 Escuela de kinesiología, Universidad de Santiago de Chile, Santiago, Chile

**Keywords:** environmental justice, green space, urban, socioeconomic status, Latin America

## Abstract

The characteristics of urban green space have context-dependent associations with socioeconomic status (SES). Latin American cities provide a unique but understudied context to assess the green space-SES associations. We measured the quantity and quality of green space as greenness from satellite-derived Normalized Difference Vegetation Index, and we modeled the relationship between greenness and SES in 371 major Latin American cities between 2000 and 2015. We found that SES was negatively associated with average greenness at city and sub-city scales, which could be explained by urbanization generally improving SES while reducing the provision of green space. About 82% of the cities and 64% of the sub-cities experienced greening or increases in greenness over time. Although with lower average greenness, cities with higher SES had greater greening; however, it was the opposite for sub-cities. We suggest that greening is more likely to take place in peripheral sub-cities where SES tends to be lower. The findings challenge the belief that places with higher SES have better access to environmental resources and amenities; instead, this relationship is context dependent.

## 1. Introduction

The urban physical environment is associated with the socioeconomic status (SES) of the residents (Jacobs *et al* 2019). Urban green space (green space hereafter), defined as vegetated outdoor areas in a city, constitutes a major component of the urban physical environment. Green space provides ecosystem services including buffering noise, supplying food, mitigating air pollution and heat, regulating water flow, treating waste water, and creating opportunities for relaxation, mental restoration, and physical activities (Gómez-Baggethun and Barton 2013, Casey *et al* 2017, Rojas-Rueda *et al* 2019). Understanding the green space-SES association helps to ensure the benefits above are shared among different populations and to promote environmental justice, a topic drawing increased attentions from the public, researchers, and local and international organizations (Wolch *et al* 2014, Jennings *et al* 2019, IUCN 2021).

Despite the importance of green space, there are SES disparities in the quantity, proximity, accessibility, quality, and temporal changes of green space. Although inconsistently, research shows that neighborhoods of low-income and marginalized race/ethnicity groups tend to have lower access to green space, and when available lower quality of green space, than other groups (Casey *et al* 2017, Rigolon *et al* 2018b, Jacobs *et al* 2019, Schüle *et al* 2019, Zhanqiang *et al* 2019). These findings support the ‘deprivation amplification’ hypothesis or the luxury effect, both suggesting that lower SES groups have limited access to health-promoting and high-quality environmental features (Macintyre 2007, Schell *et al* 2020). Studies on larger units of analysis such as cities and towns, reported mixed associations between green space and SES (Sun *et al* 2011, Yu 2015, Gwedla and Shackleton 2017, Li *et al* 2018, Rigolon *et al* 2018a).

Furthermore, studies in the US and South Africa find that higher SES neighborhoods have greater improvements in green space over time (Casey *et al* 2017, Venter *et al* 2020). In contrast, studies in China show that cities with higher per capita gross domestic product (GDP) have greater temporal reductions in green space due to urban development (Sun *et al* 2011, Yu 2015).

The variabilities in the green space-SES associations may reflect the context of the study area (Roman *et al* 2018) and differences in research designs (Jacobs *et al* 2019). Some factors shaping the context include national and local governance, citizen involvement, municipal budget, and land use regulations. Research designs may vary regarding measures of green space (e.g. quantity or proximity, private or public) and SES (e.g. income or race and ethnicity), unit of analysis (e.g. neighborhood or city), and model specifications (e.g. whether having adequate control of confounders) (Rigolon *et al* 2018b, Jacobs *et al* 2019). These differences likely lead to different associations identified.

Notwithstanding the benefits and disparities of green space, most research has been in the Global North, leaving Global South cities, particularly those in Latin America, less studied. Rigolon *et al* (2018b) identified 46 studies on the green space-SES relationship in the Global South, and only eight (17%) studied Latin American cities. In addition, these Latin American studies focused only on individual cities (Reyes Päcke and Figueroa Aldunce 2010, Romero *et al* 2012, Wright Wendel *et al* 2012, Krellenberg *et al* 2014, Fernández-Álvarez 2017) or a small set of megacities (Loret De Mola *et al* 2017). These studies report negative associations between green space and SES at the neighborhood level, which are consistent with popular findings in the Global North. Data scarcity likely limits research on a broader range of Latin American cities (Quistberg *et al* 2018). However, the associations between green space and SES in Latin America may differ from those observed in the Global North given the unique characteristics of Latin American cities, such as high urbanization rates (e.g. about 80% in 2018) (United Nations, Department of Economic and Social Affairs, & Population Division 2019), fast urbanizing of the poor, prevalence of informal settlements, and deep social inequalities (Fay 2005, Libertun De Duren 2018, Quistberg *et al* 2018).

**Figure 1.**
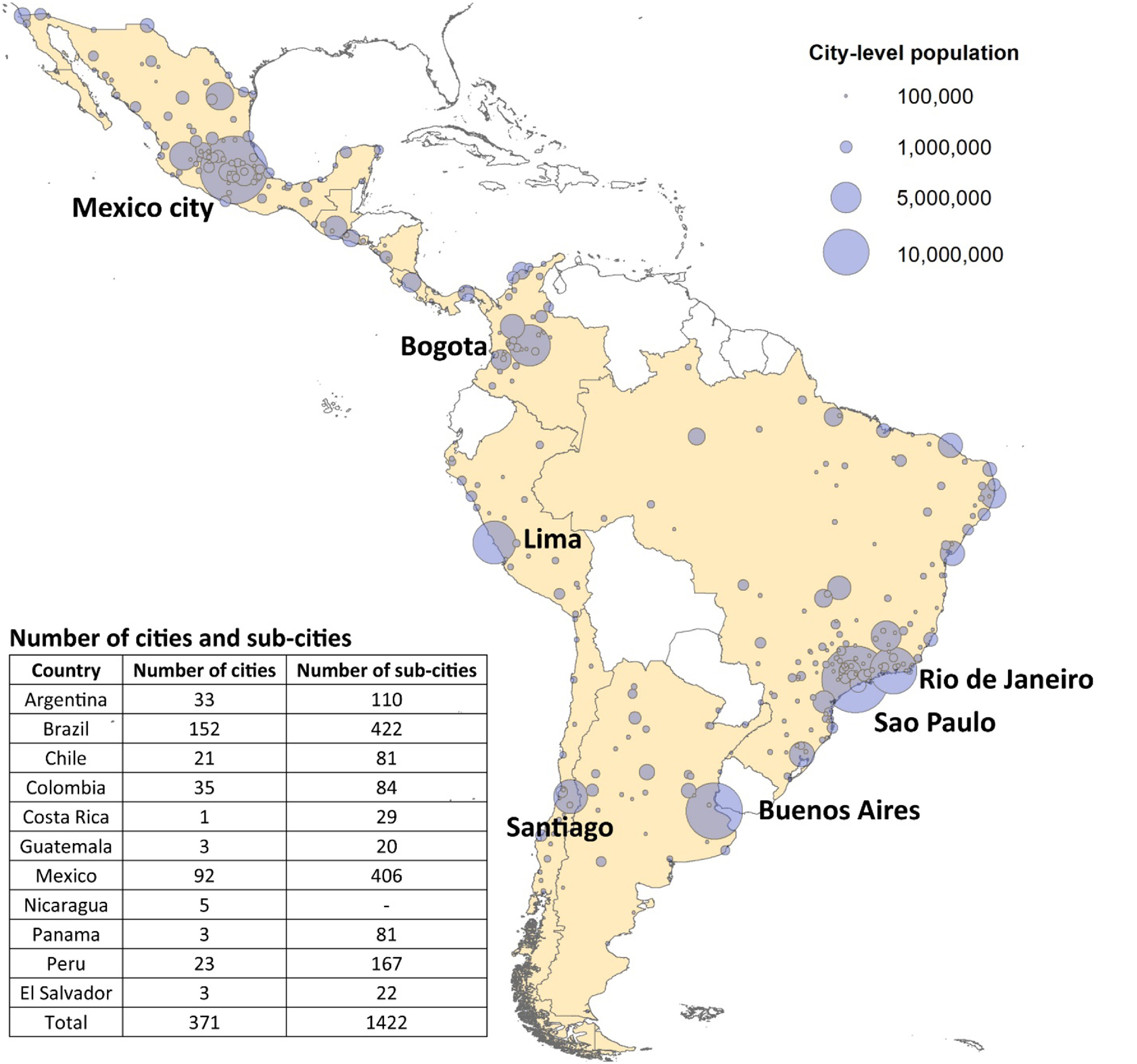
Study area, and number of cities and sub-cities by country. Reproduced from Quistberg *et al* (2018). CC BY 4.0.

We bridge a few existing knowledge gaps through this study. First, we studied the association between green space and SES for 371 major Latin American cities with more than 100 000 residents from 11 countries (figure 1), leveraging data from the Salud Urbana en América Latina (SALURBAL) project (Quistberg *et al* 2018). Second, we performed a robust research design by introducing multiple spatial scales (figure S1 available online at stacks.iop.org/ERL/16/104052/mmedia) and by stratifying the analysis by urbanization levels and climate zones. We also controlled for a comprehensive set of covariates to reduce confounding bias. We quantitatively assessed: (a) whether higher SES was associated with higher levels of greenness reflecting greater vegetation quantity and quality, averaged between 2000 and 2015; (b) whether higher SES lead to greater greening, or more increases in greenness caused by natural processes and urban development, between 2000 and 2015; (c) whether these associations above changes by alternative definitions of a city and by different urbanization levels and climates.

## 2. Methods

### 2.1. Study area and units of analysis

The study area covers 371 cities from 11 Latin American countries (figure 1), as selected by the SALURBAL study on urban health (Quistberg *et al* 2018).

Our study targeted three spatial units of analysis: city, sub-city, and the main urban cluster in a city. A city can be a single sub-city or a cluster of sub-cities sharing the same main urban cluster. A sub-city is the administrative unit, such as comunas, municipios, or similar units depending on the country, where data is relatively accessible (Quistberg *et al* 2018). A main urban cluster is the largest contiguous built-up area in a city (figure S1). This tiered approach ensures a comparable definition of ‘city’ across countries, and it is aligned with similar efforts such as the European Union-Organisation for Economic Co-operation and Development (EU-OECD) definition of functional urban areas (Dijkstra *et al* 2019). While we covered multiple cities from various countries, we were not able to focus on finer spatial units such as neighborhoods due to constraints in SES data.

### 2.2. Average and trend from greenness time series as the outcomes

We generated average greenness and greening between 2000 and 2015 using the Normalized Difference Vegetation Index (NDVI) from Moderate Resolution Imaging Spectroradiometer (MODIS) satellite (product code: MOD13Q1 V6). NDVI measures the combined effect of vegetation cover, biomass, and photosynthetic activities on a −1 to 1 scale, with values closer to 1 indicating stronger presence of vegetation (Tucker 1979). We collected 250 m resolution NDVI images every 16 days, based on best available pixels (low clouds, low view angle, and high NDVI value), from MODIS between 2000 and 2015 (Didan, n.d.). We then generated annual maximum NDVI images by compositing available images in a year. We used a global surface water dataset (Pekel *et al* 2016) to mask out water bodies, which would otherwise cause a downward bias when aggregating NDVI values. Finally, for each city, sub-city, and year we calculated the area-level median of the annual maximum NDVI values as the greenness.

We used average greenness and greening to characterize the greenness time-series in our units of analysis. Average greenness is the mean value over time. Greening is the degree by which greenness changes per year, calculated as the slope of a linear fit to the greenness time-series (equation (1)) and then rescaled to a decadal scale. Greening in this study can be caused by purposeful actions such as converting vacant lots to green space (the New York City Soil & Water Conservation District 2012), and by natural processes such as global warming (Pan *et al* 2018):

\begin{equation*}{\text{Greennes}}{{\text{s}}_{i,t}} = {\alpha _i} + {\beta _i}T + {\varepsilon _{i,t}}\end{equation*}

where ${\text{Greennes}}{{\text{s}}_{i,t}}$ is the greenness of a city *i* (city-level analysis) or sub-city *i* (sub-city analysis) at year *t*; $T$ is the number of years since 2000; ${\alpha _i}$ is the intercept and ${\beta _i}$ is the slope of the linear fit.

### 2.3. SES variables as the exposures

We measured SES by per capita GDP and a composite Social Environment Index (SEI) (Bilal *et al* 2020). Per capita GDP, averaged between 2000 and 2015, was based on a dataset by Kummu *et al* (2018) and available only at the city level. SEI is the sum of *z*-scores of the proportion of households with water in the dwelling and sewage connection, the proportion of households that are not overcrowded, and the proportion of the population aged 25 years or above completing primary education. Higher SEI indicates better social environment. We assume that per capita GDP and SEI are linked to other socioeconomic factors such as government investment for green infrastructures, tax revenue, and civic engagement, which have more direct effects on green space. SEI is available for cities and sub-cities in countries other than Nicaragua. Despite a temporal mismatch (table S10), we assumed that SEI remained relatively unchanged during our analysis timeframe.

### 2.4. Covariates

We included population density, climate zone, topography, and distance to city center that could confound the associations between average greenness, greening, and SES variables. Densely populated areas may have higher SES but limited space for green space. We obtained population density from the SALURBAL project (Quistberg *et al* 2018). Climate zone acts as a background for vegetation greenness (Ichii *et al* 2002), and it also affects economic development (Mellinger *et al* 2000). We used Koppen Climate classification (Kottek *et al* 2006) and assigned each units of analysis to its major (by area) climate zone. Topographic factors including elevation and slope affect vegetation growth (Deng *et al* 2007) and may constrain socioeconomic development (e.g. hilly cities may have a higher cost for constructing infrastructures). We derived area-level average elevation and slope from the Shuttle Radar Topography Mission digital elevation model in 2000 (Farr *et al* 2007). In the sub-city-level analysis, we included the distance between a sub-city and the city center to control for the centrality of sub-cities. Peripheral sub-cities are less urbanized, therefore likely having more green space but lower SES.

The city- and sub-city-level summary statistics of the outcomes, exposures, and covariates are in tables S1 and table S2.

### 2.5. Fixed effects model

We built fixed effects regression models between the outcomes and the SES variables. These models contain two levels to reflect the hierarchical structure of our data. For city-level analysis (equation (2)), we included the outcome, exposures, and covariates measured by cities ($i$), and we represented countries ($j$) as a set of fixed effects (${\gamma _j}$). A similar logic applies to sub-city-level analysis (equation (3)), except that we only had per capita GDP available by cities. We estimated clustered model standard errors by cities and countries. For more justifications, see section 2 of supplementary material.

\begin{align*}{y_{i,j}} &amp;= {\beta _0} + {\beta _1}{\text{GD}}{{\text{P}}_{i,j}} + {\beta _2}{\text{SE}}{{\text{I}}_{i,j}} + {\beta _3}{\text{GD}}{{\text{P}}_{i,j}} \times {\text{SE}}{{\text{I}}_{i,j}} \nonumber\\ &amp;\quad+ {X_{i,j}}\theta + {\gamma _j} + {\varepsilon _{i,j}}\end{align*}

\begin{align*}{y_{s,{ }i}} &amp;= {\beta _0} + {\beta _1}{\text{GD}}{{\text{P}}_{i,j}} + {\beta _2}{\text{SE}}{{\text{I}}_{s,{ }i}} + {\beta _3}{\text{GD}}{{\text{P}}_{i,j}} \times {\text{SE}}{{\text{I}}_{s,{ }i}} \nonumber\\ &amp;\quad+ {X_{s,{ }i}}\theta + {\tau _i} + {\varepsilon _{s,{ }i}}\end{align*}

where $i$ represents the city, $s$ represents the sub-city, and $j$ represents the country. ${ }{y_{i,j}}$ and ${y_{s,i}}$ are average greenness or greening measured at city and sub-city level, respectively. ${\text{GD}}{{\text{P}}_{i,j}}$ is per capita GDP available only at city level. ${\text{SE}}{{\text{I}}_{i,j}}$ and ${\text{SE}}{{\text{I}}_{s,{ }i}}$ are SEI measured at city and sub-city level, respectively. ${X_{i,j}}$ and ${X_{s,i}}$ represent covariates including population density, climate zone, slope, elevation, and distance to city center measured at city and sub-city levels. ${\gamma _j}$ and ${\tau _i}$ are country and city fixed effects, respectively. ${\varepsilon _{i,j}}$ and ${\varepsilon _{s,i}}$ represent random error.

In the city-level analysis, we first used per capita GDP as the exposure (model 1, table S3; model 6, table S4). We then used SEI as the exposure, controlling for per capita GDP in addition to other covariates (model 2, table S3; model 7, table S4). Finally, we tested if the relationships between the outcomes and SEI were dependent on per capita GDP by including an interaction term between per capita GDP and SEI (model 3, table S3; model 8, table S4). We followed the same logic in the sub-city level analysis, except that we excluded per capita GDP as a standalone variable as this variable was not available by sub-cities (models 4 and 5, table S3; models 9 and 10, table S4).

### 2.6. Testing the influence of analysis unit, urbanization level, and climate zone

A city includes built-up areas and undeveloped land, and consequently different types of green space (e.g. farmland versus parks). We additionally tested if results from the city-level analysis changed when we measured the outcomes and applicable covariates (i.e. population density, average elevation and slope, climate zone) by the city’s main urban cluster, a region defined by contiguous built-up areas (figure S1).

We then tested if the associations between SES, average greenness, and greening varied by urbanization levels and climate zones. We stratified the cities and sub-cities into less (bottom 50%) and more (top 50%) urbanized groups according to their percentages of built-up areas, derived from the Global Urban Footprint dataset (Esch *et al* 2018). We then stratified the cities and sub-cities into dry versus other climate groups based on Koppen Climate classification. The climate stratification allows us to test if any SES disparities in green space, or the luxury effect, are amplified in dry climate (Schell *et al* 2020). Within each group, we performed the fixed effects regression models above (equations (2) and (3)).

## 3. Results

### 3.1. SES and average greenness

**Figure 2.**
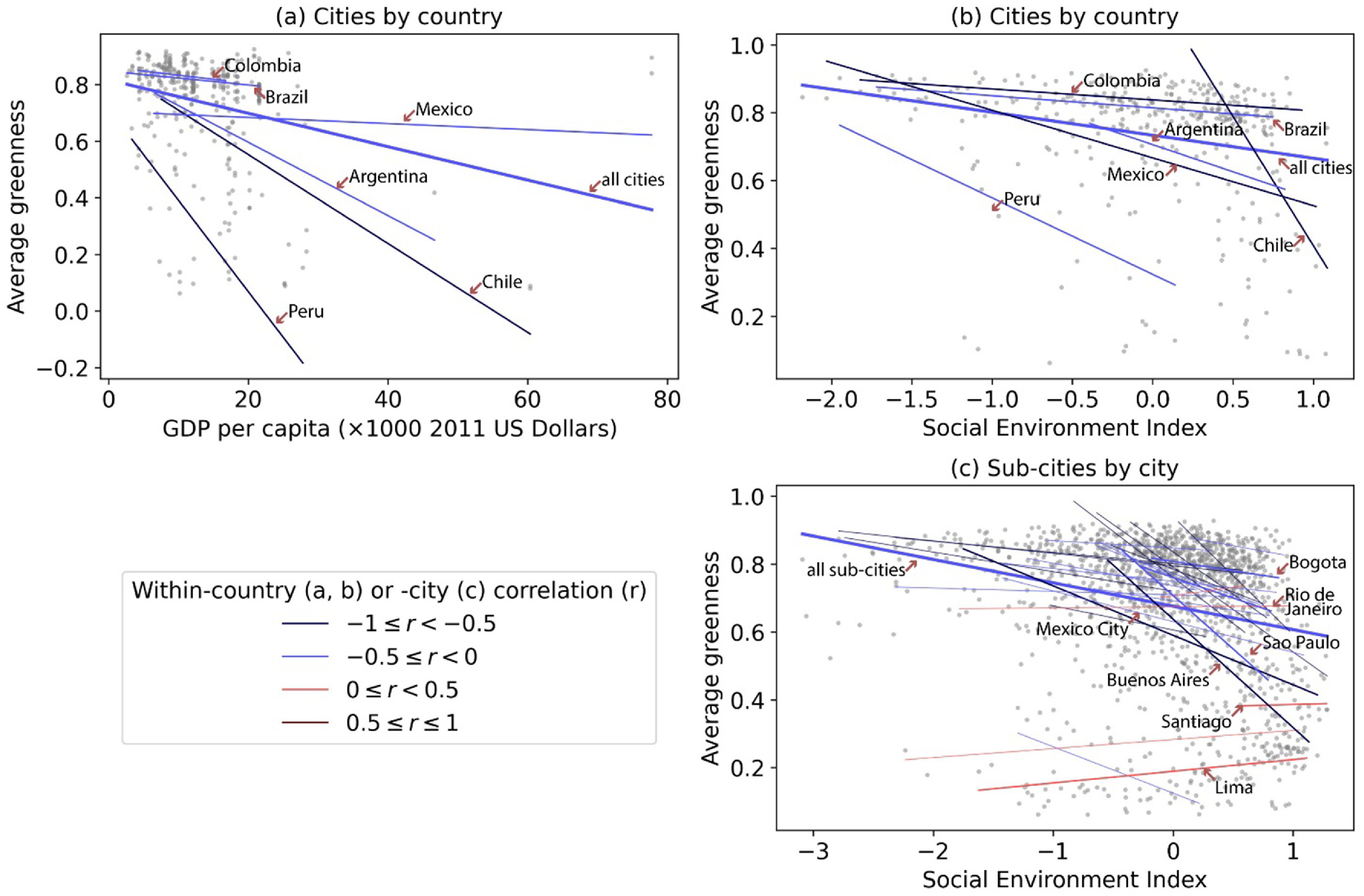
Scatter plot and correlations between SES and average greenness between 2000 and 2015 at city (a), (b) and sub-city (c) levels. The lines are with-country (a), (b) or -city (c) linear fits between SES and average greenness, with their correlations in colors. Due to smaller sample sizes, countries with less than ten cities and cities with less than ten sub-cities are excluded from the linear fit and correlation analysis.

Pearson’s correlation analysis showed that cities and sub-cities with higher SES (i.e. per capita GDP and SEI) overall exhibited lower greenness averaged between 2000 and 2015 (figure 2). This pattern was generally consistent for cities within a given country (figures 2(a) and (b)), and for sub-cities within a given city (figure 2(c)). We however found a few exceptions, including the cities of Santiago in Chile, Lima and Arequipa in Peru, and Campinas in Brazil, which showed positive associations between greenness at SES at the sub-city level.

**Figure 3.**
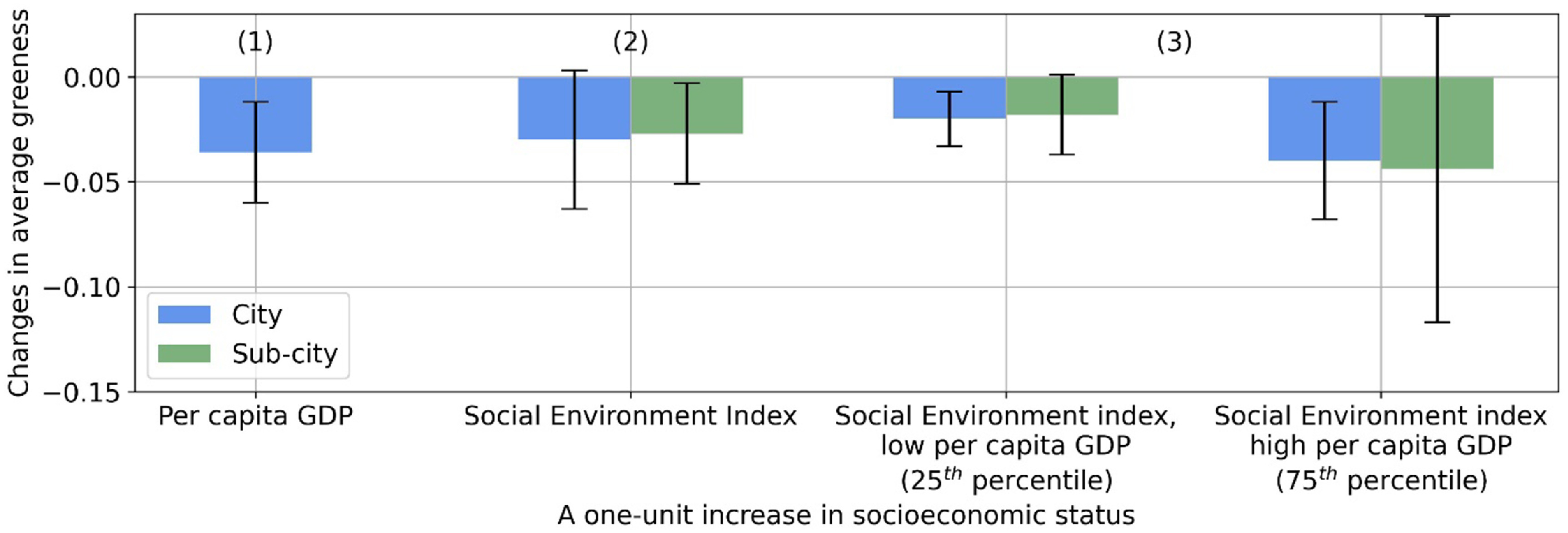
Average marginal effect (AME) of social economic status (SES) on average greenness. AME is the change in average greenness for a one-unit increase in a SES variable when holding other covariates constant, averaged across the samples. A one-unit increase corresponds to a 10 000 USD raise in per capita GDP and an interquartile range increase in Social Environment Index. The 95% confidence intervals for the AMEs are shown in error bars. AMEs for cities in columns (1)–(3) are estimated based on models (1)–(3) in table S3. AMEs for sub-cities in columns (2) and (3) are estimated based on models (4) and (5) in table S3.

Our fixed effects regression models further confirmed the overall negative association between average greenness and SES, after controlling for population density, elevation, climate zones, distance to city center (sub-city level analysis only), country- or city-fixed effects, and cluster-robust standard errors (table S3). For example, an additional 10 000 US dollars in per capita GDP was associated with a 0.036 (95% confidence interval, CI: −0.061 to −0.012) decrease in average greenness (51% of the interquartile range, IQR, of 0.070) at city level (column 1, figure 3); an IQR higher city-level SEI, controlling for per capita GDP and other covariates, was associated with a 0.030 (95% CI: −0.053–0.002) decrease in average greenness (43% of the IQR) (column 2, figure 3).

In addition, the negative association between average greenness and SEI varied by per capita GDP at the city-level, as shown by a statistically significant (*p*-value < 0.1) interaction term (model 3, table S3). For cities with lower per capita GDP (8590 US dollars, or the 25th percentile), an IQR higher SEI was associated with a 0.020 (95% CI: −0.034 to −0.007) reduction in average greenness (29% of the IQR); for cities with high per capita GDP (17 281 US dollars, or the 75th percentile), the same increase in SEI was associated with a 0.040 (95% CI: −0.069 to −0.012) reduction in average greenness (57% of the IQR) (column 3, figure 3). We found no significant dependence of SEI on per capita GDP at sub-city level (model 5, table S3).

### 3.2. SES and greening over time

About 82% of the cities and 64% of the sub-cities experienced greening, or increases in greenness, between 2000 and 2015.

**Figure 4.**
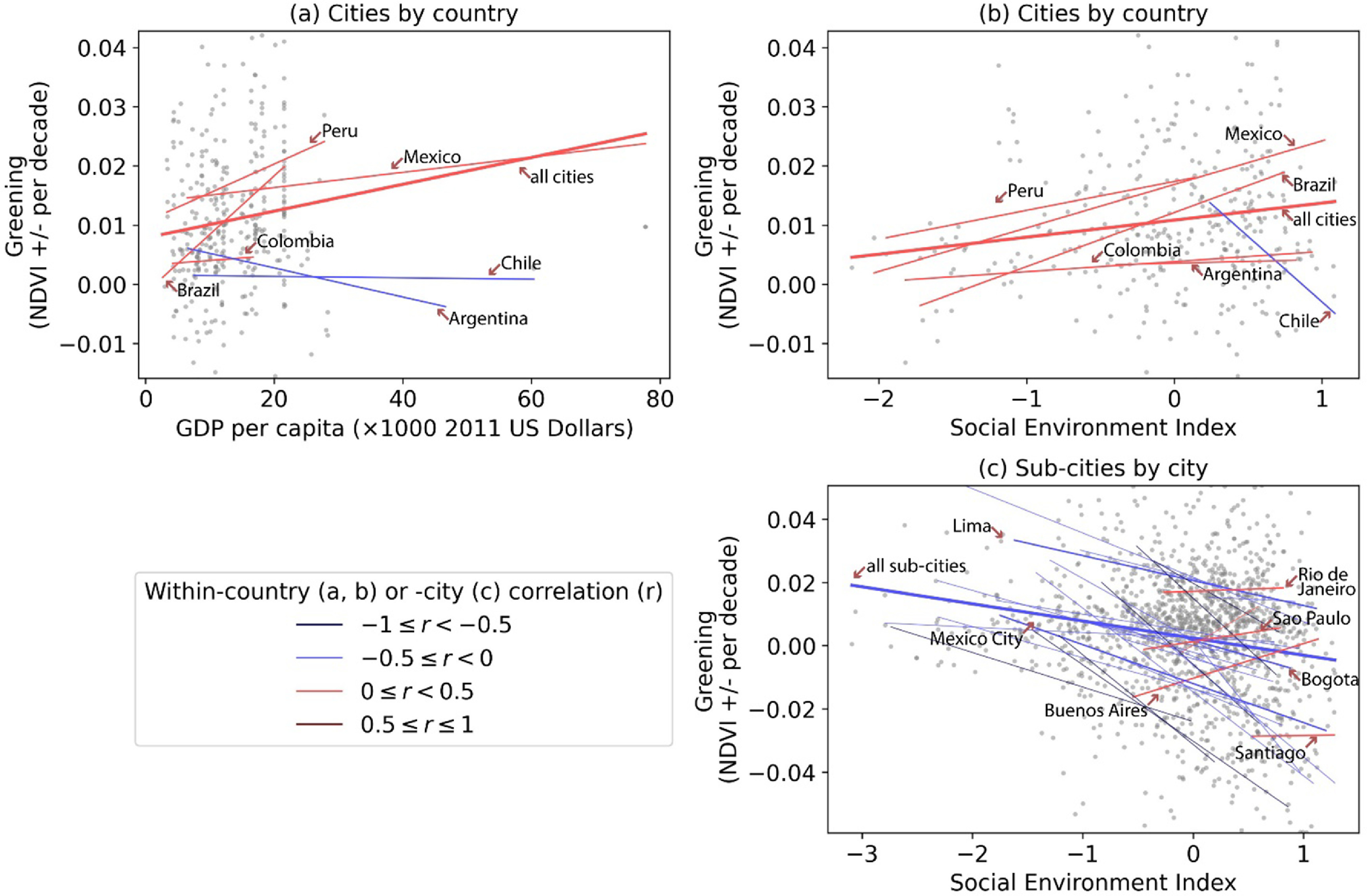
Associations between greening (changes in greenness per decade) and SES variables at city (a), (b) and sub-city (c) levels. The dots represent cities nested in countries (a), (b) or sub-cities nested in cities (c). The dash lines are country- or city-specific linear fits between greening and a SES variable, with their correlations shown in colors. Due to smaller sample sizes, countries with less than ten cities and cities with less than ten sub-cities are excluded from the linear fit and correlation analysis. For display purpose, the plots are constrained to the middle 90% range of greening.

**Figure 5.**
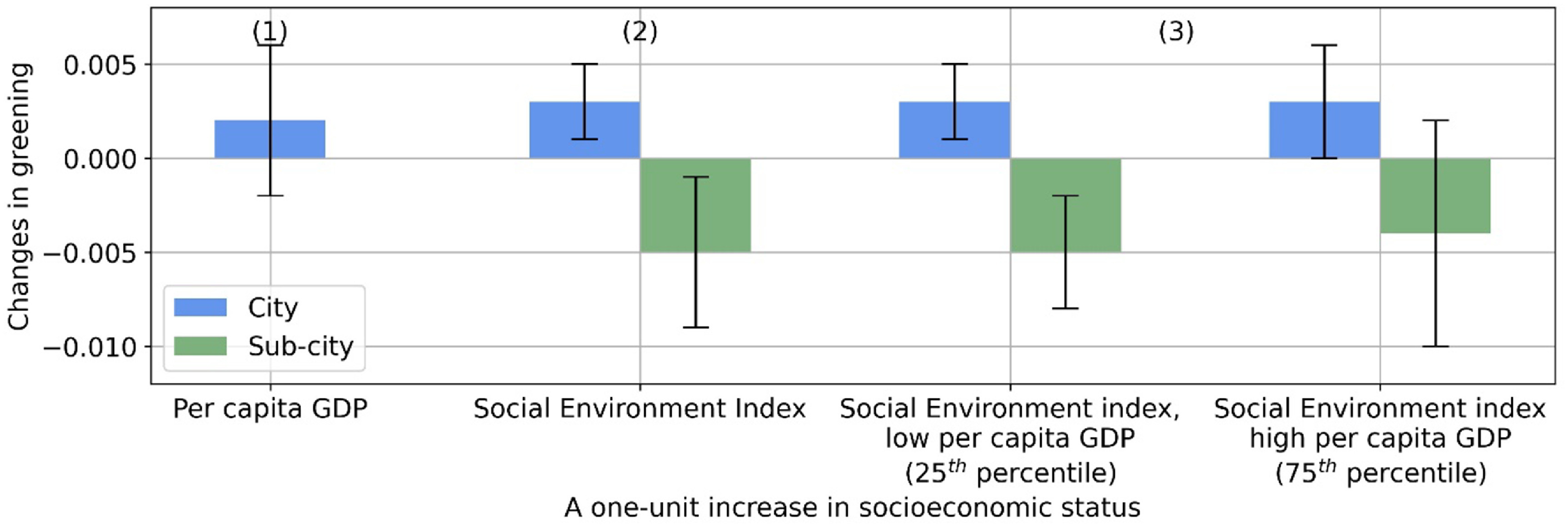
Average marginal effect (AME) of social economic status on greening (changes in greenness per decade). AME is the change in greening for a one-unit increase in a SES variable when holding other covariates constant, averaged across the samples. A one-unit increase corresponds to a 10 000 USD raise in per capita GDP and an interquartile range increase in Social Environment Index. The 95% confidence intervals for the AMEs are shown in error bars. AMEs for cities in columns (1)–(3) are estimated based on models (6)–(8) in table S4. AMEs for sub-cities in columns (2) and (3) are estimated based models (9) and (10) in table S4.

The association between SES and greening was dependent on the unit of analysis, as shown by the correlation analysis (figure 4) and regression models (table S4). In a given country, cities with higher SES were on average associated with greater greening. The association was statistically significant for SEI but not significant for per capita GDP (columns 1 and 2, figure 5). An IQR higher SEI was associated with an increase of 0.003 (95% CI: 0.001–0.006) in greening (19% of the IQR of 0.016). In addition, we find that the association between greening and SEI was independent of per capita GDP (model 8, table S4).

Sub-cities in the same city with higher SES on average had less greening, according to the correlation analysis (figure 4(c)) and regression models (model 9, table S4). An IQR higher SEI was associated with a 0.005 (95% CI: −0.008 to −0.001) decrease in greening (23% of the IQR of 0.022) (column 2, figure 5). We also found that sub-city association between greening and SEI was independent of city-level GDP (model 10, table S4).

### 3.3. The influence of analysis unit, urbanization level, and climate zone

The analysis using the main urban cluster as the unit of study (models 1–3, table S5) showed consistent results with our main, city-level analysis regarding average greenness (models 1–3, table S3). For greening, both the main analysis (models 6–8, table S4) and this analysis (models 4–6, table S5) showed similar positive associations between greening and SES variables, but the effects of SEI were not statistically significant in the main urban cluster. We also found that only 8% of main urban clusters experienced greening.

Compared with the main models, the stratified analysis by urbanization level produced similar directions of associations of the SES variables on average greenness and greening. However, some associations in the stratified analysis were not statistically significant, possibly due to smaller sample sizes (tables S6–S7).

We found that the negative associations between average greenness and SES variables in the main models (table S3) generally persisted when stratifying by dry versus other climates, with a few exceptions where these associations were not statistically significant (models 1, 9 and 10, table S8). Additionally, contrary to negative interaction between per capita GDP and SEI (model 3, table S3), we found it positive for cities in dry climate (model 6, table S8). We also found a statistically significant and negative interaction between per capita GDP and SES for sub-cities in other climates (model 8, table S8), contrary to the main model (model 5, table S3).

The associations between greening and SES variables are consistent between the main models (table S4) and models for cities and sub-cities in dry climate (models 4–6, 9 and 10, table S9). For cities and sub-cities in other climates, these associations are mostly not significant (models 1–3, 7 and 8, table S9).

## 4. Discussion and conclusion

Our findings indicated a prevalence of negative associations between average greenness and SES at city and sub-city scales across 371 major Latin American cities. We also found a positive association between greening (increases in greenness over time) and SES at the city scale. However, this association was negative in sub-cities. Furthermore, these associations with greening are more salient in dry climate, according to the stratified analysis. Since we controlled for a rich set of covariates, the relationships identified here were less likely subject to confounding bias. To our knowledge, our study is the first to study the relationship between SES, greenness, and temporal changes in greenness over a wide range of Latin American cities. Our study provides preliminary evidence to understand environmental justice in urban green space in Latin America.

We suggest that the negative association between SES and average greenness was resulted from the dominance of large cities in Latin America. Urban development generally improves socioeconomic conditions of its residents (Chen *et al* 2014), but this process also reduces green spaces through converting natural land to built-up areas (Buhaug and Urdal 2013). Compared with smaller cities, larger cities in Latin America tend to have higher SES due to their concentration of economy, population, services, health care, and educational resources (Aroca and Atienza 2016, Faraji *et al* 2016). At the same time, large cities are often more urbanized with relatively less green space, leading to the negative association between SES and average greenness that we observed.

The substantial proportion of cities (82%) and sub-cities (64%) with greening between 2000 and 2015 likely resulted from both land cover change (Liu *et al* 2020) and changes in the spectral properties of vegetation due to climate variations (Pan *et al* 2018). The positive city-level association between SES and greening might indicate that cities with better SES are more resourceful to preserve and recover green space, and that Latin American cities overall may have less demand and space for adding built-up areas due to their already high urbanization levels (about 80%). Our results also suggest that greening does not occur uniformly within a city. Only 8% of the main urban clusters experienced greening (compared to 82% of cities and 64% of sub-cities), indicating that greening is more likely take place in a city’s periphery, an area often dominated by natural or agricultural land covers. This hypothesis is also consistent with the negative sub-city-level association between SES and greening, as sub-cities with lower SES tend to locate further away from the city center.

Our findings therefore provide some evidence to examine the ‘deprivation amplification’ hypothesis or the luxury effect suggesting that low SES areas tend to have less environmental resources or lower environmental quality (Macintyre 2007, Schell *et al* 2020). Our preliminary evidence challenges these hypotheses with respect to city- and sub-city-level availability of green space (average greenness) and sub-city-level greening, but we found supportive evidence regarding city-level greening. However, our findings should be cautiously interpreted. We did not measure the type and quality of greenness. Higher greenness can be a result of carefully managed green space in some contexts, and overgrown vegetation in others. Despite representing an ‘environmental resource’ in a general sense, overgrown vegetation could introduce environmental and health risks, for example by hosting infectious disease vectors. Furthermore, the greenness metric does not reflect how people use green space.

Our findings have several implications. First, the low greenness in cities and sub-cities with high SES could reduce the net benefits of SES on public health outcomes such as lowering mortality, given that for example studies often find higher greenness associated with reduced mortality (Rojas-Rueda *et al* 2019). This is relevant for large-scale public health studies in Latin America to disentangle the interactions between health outcomes, green space, and socioeconomic conditions. Second, the positive association between greening and city-level SES suggests that purposeful urban greening should be addressed in development agendas of low SES cities, as these cities are likely in the course of urbanization and faced with greater pressure for economic and land development. Greening these low SES cities also tackles socioeconomic disparity issues in green space planning, which are likely overlooked in the region (Venter *et al* 2020). In addition, since this positive association between greening and SES is more salient in dry-climate cities, it may suggest that while better-off cities are more capable of urban greening, they should also be aware of water and climate constraints and adopt suitable vegetation and landscaping practices. In addition, if in the future climate change makes cities in other climates similar to today’s dry climate cities, the disparity in greening may also become a universal issue in the region.

Our study is subject to a few limitations. While here we used satellite-derived greenness as a proxy for green space, future studies should consider more explicit measures such as vegetation cover and biomass, public and private green spaces, natural forest and parks, and their spatial configurations. In addition, data permitting, studies should focus on finer spatial scales, for example neighborhoods, where the SES disparities of green space could be more evident and more easily interpreted by local stakeholders. The associations we identified may change if we switched to fine scales (Rigolon *et al* 2018b, Jacobs *et al* 2019) and particular types of green spaces (Chen *et al* 2017, Chen and Hu 2015, Rigolon *et al* 2018a, Wan and Su 2017). Furthermore, omitted variables, such as policy and people’s perception, may bias the associations identified. Lastly, while this study attempts to derive a ‘universal’ knowledge on green space and SES across a range of Latin American cities and countries, city- and country-specific analysis will inform more nuanced understandings about green space planning and management.

To conclude, we found a negative association between the availability of green space and SES in cities and sub-cities of Latin America. This may be attributable to that cities and sub-cities with higher SES are larger and more urbanized, therefore having limited provision of green space. We also found a positive association between greening and SES at the city level, and it changed to negative for sub-cities. The positive city-level association reaffirms the need for addressing socioeconomic disparities in developing urban green space.

## Data Availability

The data that support the findings of this study are available upon reasonable request from the authors.

## References

[erlac2a63bib1] Aroca P, Atienza M (2016). Spatial Concentration in Latin America and the Role of Institutions. Investigaciones Regionales – J. Regional Res..

[erlac2a63bib2] Bilal U (2020). Life expectancy and mortality profiles are highly heterogeneous in 363 cities of Latin America: the SALURBAL project. Nat. Med..

[erlac2a63bib3] Buhaug H, Urdal H (2013). An urbanization bomb? Population growth and social disorder in cities. Glob. Environ. Change.

[erlac2a63bib4] Casey J A, James P, Cushing L, Jesdale B M, Morello-Frosch R (2017). Race, ethnicity, income concentration and 10-year change in urban greenness in the United States. Int. J. Environ. Res. Public Health.

[erlac2a63bib5] Chen M, Zhang H, Liu W, Zhang W (2014). The global pattern of urbanization and economic growth: evidence from the last three decades. PLoS One.

[erlac2a63bib6] Chen W Y, Hu F Z Y (2015). Producing nature for public: land-based urbanization and provision of public green spaces in China. Appl. Geogr..

[erlac2a63bib7] Chen W Y, Hu F Z Y, Li X, Hua J (2017). Strategic interaction in municipal governments’ provision of public green spaces: a dynamic spatial panel data analysis in transitional China. Cities.

[erlac2a63bib8] Deng Y, Chen X, Chuvieco E, Warner T, Wilson J P (2007). Multi-scale linkages between topographic attributes and vegetation indices in a mountainous landscape. Remote Sens. Environ..

[erlac2a63bib9] Didan K (n.d.). MOD13Q1 MODIS/Terra Vegetation Indices 16-Day L3 Global 250m SIN Grid V006 [Data set]. NASA EOSDIS Land Processes DAAC.

[erlac2a63bib10] Dijkstra L, Poelman H, Veneri P (2019). The EU-OECD Definition of a Functional Urban Area.

[erlac2a63bib11] Esch T (2018). Where we live—a summary of the achievements and planned evolution of the global urban footprint. Remote Sens..

[erlac2a63bib12] Faraji S J, Qingping Z, Valinoori S, Komijani M (2016). Urban primacy in urban system of developing countries; its causes and consequences. Human (Tuzla).

[erlac2a63bib13] Farr T G (2007). The shuttle radar topography mission. Rev. Geophys..

[erlac2a63bib14] Fay M (2005). The Urban Poor in Latin America.

[erlac2a63bib15] Fernández-Álvarez R (2017). Inequitable distribution of green public space in the Mexico City: an environmental injustice case. Econ. Soc. Territorio.

[erlac2a63bib16] Gómez-Baggethun E, Barton D N (2013). Classifying and valuing ecosystem services for urban planning. Ecol. Econ..

[erlac2a63bib17] Gwedla N, Shackleton C M (2017). Population size and development history determine street tree distribution and composition within and between Eastern Cape towns, South Africa. Urban For. Urban Green..

[erlac2a63bib18] Ichii K, Kawabata A, Yamaguchi Y (2002). Global correlation analysis for NDVI and climatic variables and NDVI trends: 1982–1990. Int. J. Remote Sens..

[erlac2a63bib19] IUCN (2021). Cities and Nature: The Issues.

[erlac2a63bib20] Jacobs J, Alston L, Needham C, Backholer K, Strugnell C, Allender S, Nichols M (2019). Variation in the physical activity environment according to area‐level socio‐economic position—a systematic review. Obes. Rev..

[erlac2a63bib21] Jennings V, Browning M H E M, Rigolon A, Jennings V, Browning M H E M, Rigolon A (2019). Urban Green Spaces: Public Health and Sustainability in the United States.

[erlac2a63bib22] Kottek M, Grieser J, Beck C, Rudolf B, Rubel F (2006). World map of the Köppen-Geiger climate classification. Meteorol. Z..

[erlac2a63bib23] Krellenberg K, Welz J, Reyes-Päcke S (2014). Urban green areas and their potential for social interaction—a case study of a socio-economically mixed neighbourhood in Santiago de Chile. Habitat Int..

[erlac2a63bib24] Kummu M, Taka M, Guillaume J H A (2018). Gridded global datasets for Gross Domestic Product and Human Development Index over 1990–2015. Sci. Data.

[erlac2a63bib25] Li F, Wang X, Liu H, Li X, Zhang X, Sun Y, Wang Y (2018). Does economic development improve urban greening? Evidence from 289 cities in China using spatial regression models. Environ. Monit. Assess..

[erlac2a63bib26] Libertun De Duren N R (2018). Why there? Developers’ rationale for building social housing in the urban periphery in Latin America. Cities.

[erlac2a63bib27] Liu X (2020). High-spatiotemporal-resolution mapping of global urban change from 1985 to 2015. Nat. Sustain..

[erlac2a63bib28] Loret De Mola U (2017). On the use of hedonic price indices to understand ecosystem service provision from urban green space in five Latin American megacities. Forests.

[erlac2a63bib29] Macintyre S (2007). Deprivation amplification revisited; or, is it always true that poorer places have poorer access to resources for healthy diets and physical activity?. Int. J. Behav. Nutrition Phys. Activity.

[erlac2a63bib30] Mellinger A D, Sachs J D, Gallup J L (2000). The Oxford Handbook of Economic Geography.

[erlac2a63bib31] Pan N, Feng X, Fu B, Wang S, Ji F, Pan S (2018). Increasing global vegetation browning hidden in overall vegetation greening: insights from time-varying trends. Remote Sens. Environ..

[erlac2a63bib32] Pekel J-F, Cottam A, Gorelick N, Belward A S (2016). High-resolution mapping of global surface water and its long-term changes. Nature.

[erlac2a63bib33] Quistberg D A, SALURBAL Group (2018). Building a data platform for cross-country urban health studies: the SALURBAL study. J. Urban Health.

[erlac2a63bib34] Reyes Päcke S, Figueroa Aldunce I M (2010). Distribución, superficie y accesibilidad de las áreas verdes en Santiago de Chile. EURE.

[erlac2a63bib35] Rigolon A, Browning M, Lee K, Shin S (2018b). Access to urban green space in cities of the global south: a systematic literature review. Urban Sci..

[erlac2a63bib36] Rigolon A, Browning M, Jennings V (2018a). Inequities in quality urban park systems: an environmental justice investigation of cities in the United States. Landsc. Urban Plan..

[erlac2a63bib37] Rojas-Rueda D, Nieuwenhuijsen M J, Gascon M, Perez-Leon D, Mudu P (2019). Green spaces and mortality: a systematic review and meta-analysis of cohort studies. Lancet Planet. Health.

[erlac2a63bib38] Roman L A (2018). Human and biophysical legacies shape contemporary urban forests: a literature synthesis. Urban For. Urban Green..

[erlac2a63bib39] Romero H, Vásquez A, Fuentes C, Salgado M, Schmidt A, Banzhaf E (2012). Assessing urban environmental segregation (UES). The case of Santiago de Chile. Ecol. Indic..

[erlac2a63bib40] Schell C J, Dyson K, Fuentes T L, Roches S D, Harris N C, Miller D S, Woelfle-Erskine C A, Lambert M R (2020). The ecological and evolutionary consequences of systemic racism in urban environments. Science.

[erlac2a63bib41] Schüle S A, Hilz L K, Dreger S, Bolte G (2019). Social inequalities in environmental resources of green and blue spaces: a review of evidence in the WHO European region. Int. J. Environ. Res. Public Health.

[erlac2a63bib42] Sun J, Wang X, Chen A, Ma Y, Cui M, Piao S (2011). NDVI indicated characteristics of vegetation cover change in China’s metropolises over the last three decades. Environ. Monit. Assess..

[erlac2a63bib43] the New York City Soil & Water Conservation District (2012). Greening Vacant Lots: Planning and Implementation Strategies.

[erlac2a63bib44] Tucker C J (1979). Red and photographic infrared linear combinations for monitoring vegetation. Remote Sens. Environ..

[erlac2a63bib45] United Nations, Department of Economic and Social Affairs, & Population Division (2019). World Urbanization Prospects: The 2018 Revision.

[erlac2a63bib46] Venter Z S, Shackleton C M, Van Staden F, Selomane O, Masterson V A (2020). Green Apartheid: urban green infrastructure remains unequally distributed across income and race geographies in South Africa. Landsc. Urban Plan..

[erlac2a63bib47] Wan C, Su S (2017). China’s social deprivation: measurement, spatiotemporal pattern and urban applications. Habitat Int..

[erlac2a63bib48] Wolch J R, Byrne J, Newell J P (2014). Urban green space, public health, and environmental justice the challenge of making cities ‘just green enough’. Landscape Urban Plan..

[erlac2a63bib49] Wright Wendel H E, Zarger R K, Mihelcic J R (2012). Accessibility and usability: green space preferences, perceptions, and barriers in a rapidly urbanizing city in Latin America. Landsc. Urban Plan..

[erlac2a63bib50] Yu L G (2015). Effects of urbanization on vegetation degradation in the Yangtze River Delta of China: assessment based on SPOT-VGT NDVI. J. Urban Plan. Dev..

[erlac2a63bib51] Zhanqiang Z, Jie R, Xuan L (2019). Green infrastructure provision for environmental justice: application of the equity index in Guangzhou, China. Urban For. Urban Green..

